# Nutritional Management of Athletes with Type 1 Diabetes: A Narrative Review

**DOI:** 10.3390/nu16060907

**Published:** 2024-03-21

**Authors:** Massimiliano Cavallo, Michelantonio De Fano, Luisa Barana, Ivan Dozzani, Eleonora Bianchini, Marialucia Pellegrino, Linda Cisternino, Sara Migliarelli, Cecilia Giulietti, Roberto Pippi, Carmine Giuseppe Fanelli

**Affiliations:** 1 Department of Medicine and Surgery, University of Perugia, Unit of Internal Medicine, Terni University Hospital, Piazzale Tristano Di Joannuccio, 1, 05100 Terni, Italy; 2 Section of Endocrinology and Metabolism, Department of Medicine and Surgery, University of Perugia Medical School, 06132 Perugia, Italy; michelantonio.defano@gmail.com (M.D.F.); dozzaniivan@gmail.com (I.D.); eleonora.bianchini89@gmail.com (E.B.); marialucia.pellegrino@gmail.com (M.P.); linda.cisternino@gmail.com (L.C.); ceciliagiulietti11@gmail.com (C.G.); carmine.fanelli@unipg.it (C.G.F.); 3 Diabetology and Endocrinology, Degli Infermi New Hospital of Biella, 13875 Biella, Italy; luisa.barana@gmail.com; 4 Healthy Lifestyle Institute, C.U.R.I.A.Mo. (Centro Universitario Ricerca Interdipartimentale Attività Motoria), Department of Medicine and Surgery, University of Perugia, Via G. Bambagioni, 19, 06126 Perugia, Italy

**Keywords:** type 1 diabetes mellitus, physical activity, carbohydrates, proteins, nutritional supplements

## Abstract

Type 1 diabetes mellitus (T1DM) represents a complex clinical challenge for health systems. The autoimmune destruction of pancreatic beta cells leads to a complete lack of insulin production, exposing people to a lifelong risk of acute (DKA, coma) and chronic complications (macro and microvascular). Physical activity (PA) has widely demonstrated its efficacy in helping diabetes treatment. Nutritional management of people living with T1DM is particularly difficult. Balancing macronutrients, their effects on glycemic control, and insulin treatment represents a complex clinical challenge for the diabetologist. The effects of PA on glycemic control are largely unpredictable depending on many individual factors, such as intensity, nutrient co-ingestion, and many others. Due to this clinical complexity, we have reviewed the actual scientific literature in depth to help diabetologists, sport medicine doctors, nutritionists, and all the health figures involved in diabetes care to ameliorate both glycemic control and the nutritional status of T1DM people engaging in PA. Two electronic databases (PubMed and Scopus) were searched from their inception to January 2024. The main recommendations for carbohydrate and protein ingestion before, during, and immediately after PA are explained. Glycemic management during such activity is widely reviewed. Micronutrient needs and nutritional supplement effects are also highlighted in this paper.

## 1. Introduction

Nutritional management of people living with type 1 diabetes (T1DM) represents a multifaced clinical challenge. Due to its pathophysiology, T1DM is marked by a total absence of insulin production. The aim of insulin therapy (multi-daily injections or infusion pumps) is to mimic as much as possible glycemic response both in fasting and in a post-prandial state. Athletes affected by uncomplicated T1DM do not have different nutritional requirements from those of non-diabetic athletes, but every macronutrient (carbohydrates, proteins, and fats) affects glycemia differently. Physical activity (PA) can improve glycemic control both in type 2 diabetes and in T1DM. Nonetheless, several mechanisms can cause inadequate glycemic control around PA, tightly related to the intensity and duration of PA, the levels of circulating insulin and glucose counter-regulatory hormones, the pre-PA blood glucose level (BG), the timing and the composition of the last meal, and the location of insulin delivery [1,2]. Additionally, an individual’s physical attributes, including size, muscle mass, age, sex, fitness level, stress levels, and genetics, can also influence changes in glucose levels.

Low to moderate PA is associated with a high risk of hypoglicaemia and fear of hypoglycemia represents a strong barrier to PA in T1DM. Knowing the effects of the ingestion of different macronutrients on glycemia significantly assists people with T1DM to safely practice PA and thus improve their glycemic control. Providing this guidance is the main goal of our review. Because nutritional supplements and micronutrients are known to be useful in PA, another aim of our review is to analyze scientific knowledge about the use of supplements in people living with T1DM.

## 2. Materials and Methods

A database search was conducted in January 2024.

Our narrative review focused on various aspects related to competitive athletes with Type 1 Diabetes Mellitus (T1DM). These aspects included:

(1) the general framework of a competitive athlete with T1DM, including their muscular pathophysiology and performance.
(2) the role of carbohydrates (CHO) in managing T1DM in athletes, with a particular focus on insulin, technology, hypoglycemia prevention, and the use of CHO during different phases of training.
(3) the importance of amino acids in the diet of athletes with T1DM.
(4) the role of various micronutrients, such as creatine, selenium, caffeine, and magnesium, in managing T1DM in athletes. The search encompassed two electronic databases, PubMed and Scopus (https://pubmed.ncbi.nlm.nih.gov/ and https://www.scopus.com/search/form.uri?display=basic#basic, both accessed on 1 January 2024), spanning from their inception to January 2024. The exploration utilized the keywords “T1DM”, “Physical Activity”, “Carbohydrates”, “Proteins”, and “Nutritional Supplements”. Inclusion criteria were limited to human studies, while studies not published in English were excluded from consideration.

## 3. Results

### 3.1. General Considerations Regarding PA and EXE in People Living with T1DM

T1DM is a clinical framework characterized by insulin deficiency due to cell-mediated autoimmune destruction of pancreatic β cells. Autoimmune markers associated with T1DM include islet cell autoantibodies and antibodies targeting glutamic acid decarboxylase (GAD65), insulin, the tyrosine phosphatases islet antigen (IA-2), and IA-2 β, as well as zinc transporter 8 [3]. As of 2021, the global population of individuals with T1DM was estimated to be around 8.4 million, and this number is anticipated to undergo substantial growth in the upcoming years [4]. Insulin treatment is essential for people with T1DM, not only to limit hyperglycaemia but also to avoid other severe metabolic disturbances such as hypertriglyceridemia and diabetic ketoacidosis (DKA) [5].

PA, defined as every body movement that requires energy expenditure beyond resting expenditure to be realized, and exercise (EXE), defined as a planned, structured PA, are both beneficial to T1DM management [6].

PA and EXE increase cardiorespiratory fitness, decrease insulin resistance, and improve lipid levels and endothelial function. Further, they might contribute to avoiding micro and macrovascular complications related to diabetes mellitus, such as retinopathy, nephropathy, and major cardiovascular events [7,8].

There are different types of PA and EXE. These range from resistance to endurance to explosive to high-intensity interval training (HIIT) [9,10]. Endurance activity includes activities with a duration greater than 30 min, such as walking, dancing, and cycling, that consist of cyclical movements of large muscle groups, generally with aerobic metabolism and glucose and fat as energy sources. Resistance activity includes EXE of shorter duration utilizing free weights, weight machines, body weight, or elastic resistance bands. Explosive efforts involve ‘all-out’ exertions of muscular strength and/or speed for brief periods, typically lasting up to 10–20 s. Explosive EXE incorporates both strength and speed, generating high muscle power output, as seen in activities like sprinting, plyometrics (jumping), squats, agility drills, and heavy weightlifting. Lastly, HIIT involves near-maximal efforts during intervals in activities such as cycling, rowing, or running, lasting from 10 s to 5 min, interspersed with rests or lower-intensity efforts. HIIT is a common training strategy that optimizes cardiometabolic adaptation while minimizing the time commitment to training [9,10]. In broad terms, extended-duration aerobic EXE typically leads to a decrease in blood glucose levels, whereas higher-intensity aerobic and anaerobic EXE—especially if carried out at high intensity for less than 10 min—promotes an elevation in blood glucose levels [1]. EXE intensities play a pivotal role, with the American College of Sports Medicine (ACSM) defining moderate-intensity EXE as falling within the range of 40% to 60% of maximal capacity, while high-intensity EXE surpasses 65% of maximal capacity [11].

Several studies have shown the positive impact of PA and EXE on T1DM management.

In a cross-sectional multicentre study [12], 18,028 patients with T1DM (>18 to <80 years of age) were categorized based on their self-reported frequency of PA. The stratification included three groups: PA0 (inactive), PA1 (one to two times per week), and PA2 (more than two times per week). Multivariable regression models highlighted a significant reduction in glycated haemoglobin (HbA1c), daily insulin dosage, and Body Mass Index (BMI) for the PA2 group. Also, rates for severe hypoglycaemic episodes, comas, and DKA were lower in the PA2 group. Furthermore, multivariable regression models, applied for cardiovascular risk factors, showed a favourable impact of intense PA in reducing the values of diastolic blood pressure and the prevalence of dyslipidemia, retinopathy, and microalbuminuria.

In another observational cross-sectional study, involving 130 adults with T1DM having a mean age of 33.9 years and a disease duration of 16.5 years, the association between the intensity of PA (expressed in metabolic equivalent units or METs) and metabolic control was examined [13]. Participants were categorized into three subgroups based on their level of PA in the preceding month, according to Pate et al. [14]: light activity (<3 METs), moderate activity (3–6 METs), or intense activity (>6 METs). The results showed that performing more than 150 min of intense PA is associated with significant improvements in HbA1c values [13].

Finally, two different meta-analyses of randomized control trials (RCTs) confirmed the positive effects of EXE in T1DM. The first, which included 24 randomized control trials (published between 1970 and 2005) and involved 1435 individuals with T1DM, demonstrated that EXE was associated with an improvement in HbA1c of 0.33% [15]. The second, the most recent, evaluated the effects of EXE in 509 youths with T1DM (involved in 14 studies), and also examined the impact of different types of intervention. The study demonstrated that programs extending beyond 24 weeks, incorporating at least 60 min per session of high-intensity EXE, could potentially function as a supportive therapy for sustaining optimal metabolic control in youths with T1DM [16].

As can be seen, PA and EXE have different glycaemic responses in those with T1DM, so it is crucial to program timing, type, intensities, frequencies, and duration of PA, according to pharmacological and dietary treatment, to better manage blood glucose levels. EXE guidelines for type 2 diabetes are clear, but those for T1DM are complex due to glycaemic control and adherence issues. A personalized approach is needed for maximum benefit [17].

The American Diabetes Association (ADA) [5], the World Health Organization (WHO) [17], and the ACSM [11] align in emphasizing the importance of regular aerobic EXE for maintaining overall health. The recommended duration for most individuals is a minimum of 150 min of aerobic moderate activity per week or 75 min of vigorous activity per week. This activity should be distributed across at least 3 days, with no more than two consecutive days off. Younger, physically fit individuals may also consider HIIT as an alternative. Moreover, adults with diabetes are advised to engage in 2–3 sessions of resistance EXE per week, on non-consecutive days [6,18]. It is recommended that EXE be supervised by an EXE specialist, as this has proven to be more effective than unsupervised training [19,20]. For older adults, flexibility and balance EXE should be done 2–3 times per week. EXE is also recommended for patients with T1DM, even if their glycaemic response to EXE is highly variable [1]. Finally, it is important to incorporate spontaneous PA throughout the day to counteract sedentary time [18].

The utilization of substrate in humans during EXE tends to vary based on the intensity of the EXE. In simple terms, during moderate to intense EXE, the body shifts from using fat as the main fuel to relying on carbohydrates [21]. It is important to note that when skeletal muscle contracts, it requires energy that is supplied by Adenosine triphosphate (ATP). Phosphocreatine immediately resynthesizes this ATP. However, the limited stores of phosphocreatine require the catabolism of other fuel sources (such as lipids and carbohydrates) to resynthesize ATP for EXE events of shorter duration. Monaco et al. [22] have highlighted that T1DM negatively affects the mitochondrial function and the morphology, metabolism, function, and repair of skeletal muscle and that this may contribute to glycemia alterations linked with a slower phosphocreatine recovery time [23].

For higher-intensity EXE, free fatty acid (FFA) utilization decreases and muscles rely only on glycogen and glucose for energy. Riddell et al. [10] elucidated the impact of insulin treatment on the flow of glucose from the liver to the muscles during EXE, which can give rise to hypo- or hyperglycemia. Elevated insulin levels restrict hepatic glucose mobilization and enhance muscle glucose disposal, potentially leading to hypoglycemia. In contrast, insufficient insulin production results in increased blood glucose levels, leading to hyperglycemia. This happens because the amount of glucose produced by the body is more than what it can use [20]. For EXE of moderate intensity, glucose and lipids are equally used. As intensity decreases, FFA becomes the primary source and carbohydrate use declines. Although intensive insulin therapy in T1DM has been shown to increase body fat stores and weight [21,22], exercising with high insulin levels suppresses fat oxidation compared to basal insulin concentration [23]. In people with T1DM, during EXE, insulin levels cannot be immediately reduced, which can cause hyperinsulinemia. This condition can suppress the process of fat oxidation and lipolysis while increasing glucose utilization, thereby increasing the risk of hypoglycaemia. In the case of intense EXE, post-EXE hyperglycaemia can be worsened due to the inability to increase insulin release and distribute it into the portal [1].

Approximately 5% of individuals with T1DM are competitive athletes, actively participating in high-performance sports. However, the daily management of the condition, particularly during training and competition preparation, poses challenges such as monitoring glucose levels, counting carbohydrates/macronutrients, adjusting insulin dosages, and managing stress or illness days. Handling these aspects remains a demanding task. Ongoing research is progressively delving into the distinctive physiology of high-level athletes with T1DM, concurrently exploring the potential benefits of new insulin analogs and other therapeutic agents/technologies to enhance their glycemic management [10].

### 3.2. Nutritional Management of People Living with T1DM Undergoing PA: Focus on Carbohydrates

#### 3.2.1. General Carbohydrates Requirements for Performance

Athletes affected by uncomplicated T1DM do not have different nutritional requirements from those of non-diabetic athletes; regarding performance enhancement [24], they need to modify their intake considering glucose levels and the individual’s insulin management plan. Athletes with T1DM do not have specific macronutrient recommendations, but guidance is drawn from a consensus among athletes and guidelines for nutritional requirements and diabetes management in individuals with diabetes [25].

What’s more, athletes—with and without diabetes—have the same nutritional requirements of normal people, according to the principles of the Mediterranean diet: 45–65% carbohydrate (CHO), 20–35% fat, and 10–35% protein, albeit that for T1DM athletes total energy requirements, macro, and micronutrients distribution should be individualized based on body weight, insulin therapy, glycaemic control, and specific EXE and athletic goals [26].

Carbohydrate requirements play a pivotal role in enhancing athletic performance. They serve as fuel for the brain and central nervous system and act as a versatile substrate for muscular work, supporting EXE across a broad range of intensities through utilization via both anaerobic and oxidative pathways [27,28]. While carbohydrate stores are relatively limited, they can be acutely manipulated daily to ensure high carbohydrate availability during performance [27]. It’s important to note that athletes with T1DM can maintain normal levels of muscle and liver glycogen content only when adequately nourished, administer insulin, and maintain good glycemic control [10,28].

Daily CHO intake guidelines for athletes are typically expressed per kilogram of body mass, ranging from 3–12 g/kg. For athletes engaged in skill-based activities and recreational pursuits, 3 g/kg is likely sufficient. Moderate-intensity EXE lasting approximately 1 h per day suggests a target CHO intake range of 5 to 7 g/kg. Endurance athletes involved in moderate-to-high intensity training for 1–3 h per day are advised to aim for 6–10 g/kg. Those participating in more extreme and longer-duration EXE (more than 4 h per day) may require a CHO intake in the range of 8–12 g/kg [27].

Certainly, in T1DM athletes, it’s crucial to differentiate between carbohydrate needs for performance and carbohydrate intake necessary for hypoglycemia prevention. For instance, 15 g/h of carbohydrates may be suitable for preventing hypoglycemia but might not be sufficient for optimizing endurance performance [1]. On the other hand, elevated carbohydrate supplementation (up to 75 g/h) is feasible during prolonged activities such as marathons and other endurance-type races without negatively impacting glycemia, provided the insulin dose is titrated appropriately [1,10]. In fact, carbohydrate requirements will alter insulin management strategies and vice versa. In this context, the timing of CHO consumption is crucial and can make a difference in enhancing performance.

Before an athletic event, CHO intake can rise from 7–12 g/kg per 24 h to 10–12 g/kg per 36–48 h [3]. “Carbohydrate loading” refers to the consumption of extra CHO in the days preceding an event to maximize muscle glycogen stores, particularly for activities lasting longer than 90 min [3]. There is no consensus about efficacy in athletes affected by T1DM [23,28,29,30].

The timing of the pre-event meal is individualized, although a meal consumed 2–4 h before the event is generally suitable for most athletes. For individuals with T1DM, there can be benefits to consuming the pre-event meal at least 4 h before the event. This allows the athlete to start the event with low circulating insulin levels, which may be advantageous in endurance events and for individuals prone to hypoglycemia [10].

Selecting the appropriate type of carbohydrate for EXE is crucial and relies on the glycemic index (GI) of foods. Consuming low-GI foods before EXE may sustain carbohydrate availability and help maintain euglycemia. Conversely, gels, drinks, or snacks with a high GI deliver carbohydrates rapidly, elevating blood glucose concentrations and aiding in recovery. In the context of T1DM, these high-GI options can also serve to treat or prevent hypoglycemia [31,32].

Furthermore, the choice of a low-GI CHO has the potential to minimize insulin requirements. Lower circulating insulin levels may help reduce the risk of hypoglycemia both during and after EXE, especially when insulin sensitivity is enhanced [28,31].

On the other hand, the pre-EXE blood glucose level (BG) can influence food choice, according to its GI index: with moderate/elevated pre-EXE BG, low-GI foods can be ingested earlier during activities without affecting glycaemia, whereas, when the pre-EXE BG is low, high-GI CHO foods such as energy drinks and energy gels may need to be taken immediately [28,31]. Clearly, CHO needs have been shown to vary according to the levels of circulating insulin from the last insulin administration [33,34].

During EXE, generally, CHO supplementation is not necessary for EXE < 45 min [27], even if 0.1–0.3 g/kg may be useful to prevent hypoglycaemia in diabetics [1]. During endurance or extra endurance PA, 30–60 g/kg (up to 90 g/kg in case of extra endurance or performance > 90 min) are recommended [1,10]. High-glycaemic-index food and fast-acting CHOs are preferred [35], and the choice of CHOs should aim to maximize CHO oxidation while preventing gastrointestinal problems. For instance, a mixture of glucose and fructose has demonstrated a high oxidation rate (approximately 1.7 g/min), while isomaltulose and fructose oxidize at lower rates (0.6 g/min) and are not routinely recommended for ingestion during EXE [36]. In contrast, consumption of a carbohydrate with a low GI (isomaltose) 2 h before a high-intensity run in adults with T1DM showed better blood glucose responses during EXE than did the consumption of a carbohydrate with a high GI (dextrose) [37]. Additionally, it is recommended to use a combination of carbohydrates that utilize different intestinal digestion and transport systems, especially for high carbohydrate requirements (>70 g), known as “multiple transportable carbohydrates”. This approach aims to enhance total intestinal absorption [38].

After prolonged aerobic activities, or endurance competition, a CHO intake of 1.0–1.2 g/kg BM/h for the first 4 h is required to replace glycogen stores in the immediate post-EXE period (0–4 h) [1,27,28]. The combined ingestion of a small amount of protein (0.2–0.4 g/kg BM/h) with smaller amounts of CHO (0.8 g/kg BM/h) results in similar muscle glycogen synthesis compared to larger amounts of CHO ingested alone [10]. The athlete with T1DM should consider administering a small insulin bolus with the post-EXE CHO to facilitate rapid glycogen synthesis [1,10]. What’s more, in T1DM, low-GI post-EXE foods can preserve carbohydrate availability and stable blood glucose levels, together with an individualized reduction in basal insulin rate in the post-EXE period [33]. This safeguards also against postprandial hyperglycaemia and inflammation for approximately 8 h post-EXE. The prevention of late nocturnal hypoglycaemia could be facilitated by introducing a bedtime snack containing protein in combination with low-GI carbohydrates and fat after intense or extended PA [25,26,39].

#### 3.2.2. Glycemic Management of PA

Dysglicemia can be prevented both by dietary approach and modulating insulin injection. Next to dietary management, technology strategies in T1DM therapy—such as glucose monitoring systems (CGM) and insulin infusion pumps—play a key role [1,26].

CGM devices consist of an embedded sensor, attached to the arm or abdomen, that measures glucose in the interstitial fluid in real time (rt-CGM) or intermittently, on demand [39,40,41]. They also inform as to the direction, magnitude, duration, frequency, and rate of change of glycaemia; data is transmitted to a receiver (e.g., a cell phone or other reader device) [42]. These systems can be used as effective tools to help indicate when carbohydrate intake should be initiated or avoided [43,44]. The data report shows overall glycemic control, expressed by a standardized glucose target, defined as time in range (TIR), time below range, and time above range.

The lag time, however, between the glucose value in the vasculature and interstitial fluid can influence sensor glucose measurement accuracy, especially in situations of rapid glucose changes [45,46]. Accuracy of measurements can also be influenced by EXE type, sensor site, vasoconstriction, changes in blood flow rate, and body temperature, and acidity can potentially impact interstitial glucose-sensing accuracy [47,48]. Nevertheless, data from several studies assessing the accuracy of continuous glucose monitoring (CGM) during EXE have been promising. The overall mean of all Mean Absolute Relative Differences (MARDs), a measure used for accuracy assessment, during different types of EXE in individuals with T1DM is reported as 13.63% [49].

Continuous subcutaneous insulin infusion (CSII) involves a programmable pump that continuously delivers rapid-acting insulin via an infusion set inserted subcutaneously. This system offers higher precision and flexibility in adjusting insulin basal rates and boluses [50]. The basal insulin infusion rate can be varied at least hourly, and temporary adjustments, either up or down by a fixed percentage, can be made [51]. The combination of rt-CGM with CSII, has significantly impacted glucose profiling and diabetes management. Recent technological advances, such as automated insulin delivery (AID) systems or closed-loop systems, also known as artificial pancreas systems, automatically adjust some aspects of insulin dosing based on an algorithm’s response to continuous data from a glucose sensor and information from an insulin pump, along with additional data [26]. The risk and burden of EXE-induced dysglycemia may be addressed with closed-loop systems that modulate insulin delivery in a glucose-responsive manner autonomously [1,26,30,52].

Despite their robustness, wearing insulin pumps may not be practical for certain contact sports, and athletes face the risk of cannula displacement. If not detected early enough, this displacement can lead to hyperglycemia and ketoacidosis [26]. Some reports suggest that newer patch pumps can be placed in body locations where they are better protected from damage (e.g., the inner thigh). However, these locations may not always be ideal for insulin delivery.

#### 3.2.3. Glycemic Targets before and during PA

The appropriate blood glucose concentration at the beginning of EXE should be tailored to the individual and depends on various factors, including the type and duration of PA or the use of devices (CGM or CSII or both) [40].

According to the Consensus Statement by Riddel et al. for EXE management in T1DM [1], a reasonable glucose concentration starting range for most patients engaging in aerobic EXE lasting up to an hour is 7–10 mmol/L (~126–180 mg/dL). For anaerobic EXE and HIIT [10], a lower starting glucose concentration of ~5–7 mmol/L (~90–126 mg/dL) can be considered. PA should not be initiated with elevated blood or urinary ketone bodies or severe hypoglycemia (≤2.8 mmol/L or ~50 mg/dL) within the previous 24 h.

During PA, the target should rise between 126 and 180 mg/dl and CHO consumption should be recommended when glycaemic values are about 126 accompanied by a horizontal trend arrow. EXE should be suspended for values <70 or >270 mg/dl (if insulin correction is applied, the regular correction factor might be reduced by up to 50% [1]. In general, it is recommended that athletes strive for more than 70% Time in Range (TIR) within the range of 3.9–10.0 mmol/l, with a target of 75% TIR during competition [1]. Additionally, it is advised to reduce glycemic variability, aiming for a coefficient of variation of ≤36% for continuous glucose monitoring (CGM) values. Values above this threshold appear to correlate with an increased risk of hypoglycemia [53]. When using an rt-CGM with an alarm, people T1DM diabetes suffered significantly fewer episodes of hypoglycaemia during EXE. The alarm was used to trigger carbohydrate intake to avoid incipient hypoglycaemia, according to BG and its rate of change (arrow) [43,45,49].

The position Statement of the European Association for the Study of Diabetes (EASD) and the International Society for Paediatric and Adolescent Diabetes (ISPAD) [40] recommend safe glycaemic range and specific carbohydrate consumption in response to sensor glucose value together with the corresponding trend arrow prior to, during, or after EXE, with regard to different groups categorized by hypoglycaemia risk [43].

Due to the inability to immediately lower insulin levels at the onset of EXE, individuals with T1DM often experience hyperinsulinemia during PA [34]. To counteract relative hyperinsulinemia during prolonged aerobic EXE, adjustments can be made through reductions in basal and/or prandial insulin doses or by increasing CHO intake [33]. Trained individuals with T1DM may experience greater reductions in blood glucose concentrations during aerobic EXE compared to individuals with reduced physical fitness. This difference could be attributed to the higher overall work rate in those who are more aerobically conditioned [54]. Hypoglycaemia develops in most athletes within 30–45 min of PA initiation [55]. Interestingly, HIIT sprint in T1DM promotes increased oxidative capacity of skeletal muscle and attenuates the rates of glycogen breakdown, theoretically offering protection against post-EXE hypoglycemia. Additionally, resistance EXE is associated with better glucose stability, although it may cause a modest rise in glycemia [25].

When EXE is scheduled within 2–3 h of a meal, adjusting the corresponding dose of pre-EXE insulin is crucial [40]. Bolus dose reductions require careful planning and are likely suitable for EXE with a predictable intensity performed within 2–3 h after a meal [1,40]. Nonetheless, reducing the bolus insulin dose by as much as 75% has been deemed safe and effective, without increasing ketone production during EXE [38]. Combining a 75% reduction of the bolus with the ingestion of a snack or meal with a low glycemic index has been shown to reduce the risk of hyperglycemia [56]. However, this approach will not protect against hypoglycemia if EXE is performed an hour or more after consuming the snack.

EXE may enhance the rate of absorption of subcutaneously delivered insulin, increasing insulin action soon after bolus administration [34]. It is recommended to administer insulin in an area that is not actively engaged in muscle contraction [40].

For individuals using multiple daily injections (MDI), the basal insulin dose can be reduced by 20–50% before EXE to mitigate hypoglycaemia risk [1,10,33]. For CSII users, insulin infusion rates should be reduced between 50% and 100% one to two hours before EXE, as circulating insulin levels at the start of EXE are a predictor of hypoglycemia during EXE [57]. An 80% reduction in basal insulin at the onset of EXE helps mitigate hyperglycemia after EXE more effectively than basal insulin suspension and appears to be associated with a reduced risk of hypoglycemia during and after the activity [58]. For prolonged aerobic EXE, insulin delivery can be suspended at last for less than 2 h to limit the risk of compromised glycemic control and ketosis [40]. Each commercially available Automated Insulin Delivery (AID) system has the option of activating an EXE or activity glucose target in anticipation of EXE or PA. The purpose of an “EXE target” is to increase glucose levels and maintain a higher blood glucose target during EXE by adjusting the insulin delivery algorithm. It should be set well in advance of aerobic EXE, 90–120 min prior PA, to reduce hypoglycaemia risk [57]. For unplanned EXE, the target must be activated near the beginning, so less basal insulin will be delivered during the activity. A target is not necessary for anaerobic or short aerobic performance (<30′) [39]. For aerobic or mixed aerobic–anaerobic EXE, it is necessary to reduce the bolus amount by 0–25% 1–3 h prior (maybe up to 75% if prolonged EXE is anticipated) [58].

#### 3.2.4. Glycemic Management after PA

For athletes with T1DM, it is crucial to rapidly and adequately replenish muscle and liver glycogen stores to help prevent late-onset hypoglycaemic [33,34] or euglycemic ketosis during EXE recovery [1].

Anaerobic PA and HIIT sessions are typically associated with a smaller decrease in blood glucose levels and a modest rise in glycemia. This is due to increased concentrations of counterregulatory hormones and various metabolites that restrict glucose disposal [55]. Hyperglycemia commonly occurs in patients after intense EXE, particularly if insulin concentrations are reduced [34].

Low-to-moderate intensity continuous PA is associated with an increased risk of PA-induced hypoglycaemia during PA and for up to 11 h after PA [50], with the greatest risk of nocturnal hypoglycaemia occurring after afternoon activity [54,59]. Different metabolic pathways are involved in hypoglycaemia onset. At the very beginning of PA, hypoglycaemia can occur via the activation of α-adrenergic autonomic afferent pancreatic fibres [60], a condition known as hypoglycaemia-associated autonomic failure (HAAF) [59,61] irrespective of the reduction of basal insulin infusion rates. As a result, individuals with T1DM typically require an increased carbohydrate intake or an insulin dose reduction, or both, before commencing aerobic EXE. Most hypoglycaemia might otherwise be induced due to inadequate insulin administration, such as inadequate reduction of insulin infusion rates by insulin infusion pumps. After aerobic EXE, glucose uptake into muscle decreases immediately but peripheral glucose uptake remains elevated to help replenish glycogen stores via an increase in plasma-membrane-associated GLUT4 within 2 h post-EXE, and through increased insulin sensitivity in the following hours [54]. Elevated insulin sensitivity after EXE, and possibly a blunting of glucose counter-regulation, appear to place individuals at risk for at least 12 h [59,60], or 24 h in recovery from prolonged or high-intensity EXE. Late postprandial hypoglycemia (>4 h after a meal) following aerobic EXE is driven partly by circulating basal insulin concentrations. Additionally, sometimes overcorrection of hyperglycemia occurs after PA, via repeated insulin dose administration, resulting in an increased risk of severe late-onset hypoglycemia, which could even be fatal [59]. The overnight use of a CGM system with alarms and education in the interpretation of glycemic trends and arrows is essential for T1DM athletes [41,43,49]. To avoid overnight or late-onset hypoglycaemia, a bedtime snack or a prolonged “EXE-target” during the night should be recommended [57]. Nonetheless, Campbell et al. have shown that, despite consuming sufficient carbohydrates for recovery post-EXE and a low-GI snack bedtime, hypoglycaemia was still encountered late after EXE in the early hours of the morning when DMT1 is treated on a basal–bolus regimen [56].

Notably, patients who take multiple insulin injections may experience hypoglycemia due to relative hyperinsulinemia, as they cannot decrease their baseline insulin concentrations when taking a long-acting component [62]. In contrast, reducing long-acting basal and prandial insulin concentrations before EXE decreases the risk of hypoglycemia during and after the activity, but may lead to hyperglycemia at other times of the day [33]. Therefore, outcomes may differ in patients using continuous subcutaneous insulin infusion therapy. CSII provides better flexibility in basal insulin adjustments and management of EXE-associated hyper- or hypoglycemia than other insulin delivery methods in the management of early-onset and late-onset hypoglycemia after EXE [25,57,63]. When using a CSII or AID, a higher “EXE/activity target” should be maintained for 1–2 h in recovery; the insulin basal rate should be reduced on average 20% overnight following an EXE session [64] and bolus must be reduced up to 50% at the post-EXE meal (the starting plan for post-EXE meal insulin is a 25% bolus reduction, irrespective of the type of EXE) [40].

The main results of the previous sections are shown in Table 1.

### 3.3. Nutritional Management of People Living with T1DM Engaging in PA: Focus on Proteins and Fats

#### 3.3.1. Effects of Proteins and Fats on Glycaemia in T1DM People

Proteins and fats are two of the main macronutrients of human nutrition. A balanced nutritional plan includes a correct combination of these three components, including fiber and water as other essential parts. Both proteins and fats affect glycemia indirectly. In fact, while carbohydrate consumption leads directly to an increase in glycemia in a short time (within 2–3 h), proteins and fats increase glycemia more slowly (up to 8 h) due to their capability to induce gluconeogenesis, a biochemical pathway to produce glucose (the main human metabolic fuel) in the absence of carbohydrates transforming some aminoamides (from proteolysis) and glycerol (from lipolysis). Predicting how a balanced meal could interfere with glucose metabolism is challenging. In people living with T1DM, due to the complete absence of endogenous insulin production, there is an upregulation of gluconeogenesis and hepatic glycemic output with a consequent tendency to hyperglycemia. Branched-chain amino acids (BCAA) leucine, isoleucine, and valine, deriving from proteolysis, seem to deeply affect glycemia beyond gluconeogenesis promoting insulin resistance [65], impairing insulin signaling with a constant stimulation of the mTOR pathway [66], and promoting beta-cell intrinsic dysfunction affecting its integrity. Recent studies reveal that a reduction in BCAA consumption could lead to better post-prandial glycemic control while also ameliorating insulin sensitivity [67,68,69]. In fact, exogenous insulin administration tries to mimic one of the most complex physiological pathways, trying, in normal conditions, to keep euglycemia both during fasting and in the post-prandial state. Carbo-counting represents a very important tool for people living with T1DM, but it doesn’t consider at all the unavoidable presence in a balanced meal of proteins and fats. Rapid-acting insulin analogues maintain their hypoglycemic activity for a few hours (2 or 3 h), counterbalancing the carbohydrate content of a meal well, but are not capable affecting other macronutrients in a perfect way. This represents a complex clinical challenge for diabetologists, partially mitigated by continuous subcutaneous delivery systems of insulin integrated with continuous glycemic monitoring systems that automatically correct glycemia following actual glycemic trends. A deep knowledge of the glycemic effects of proteins and fats is vital to help people living with T1DM to better control their glycemic metabolism and avoid both hyperglycemia and hypoglycemia.

#### 3.3.2. Protein and Fat Consumption in People Living with T1DM Engaging in PA

Carbohydrates are the main fuel during anaerobic EXE and limited during aerobic one. Prolonged aerobic EXE needs mainly lipids as fuel. Aerobic activity is generally characterized by an increase in lipid oxidation. Hyperinsulinemia, which could take place in people affected by T1DM mellitus due to exogenous insulin administration, could affect this mechanism by interfering with lipolysis and fat oxidation. In a typical physiological state, higher PA intensities lead to an increase in glycolysis and lactate production with a reduction in fat oxidation. In T1DM people, on the other hand, lipolysis is enhanced by the presence of a great amount of high-affinity beta-adrenoceptors in adipose cells. If carbohydrate consumption is limited in T1DM people, fat oxidation is further promoted with a concrete risk of impairment during high-intensity PA but with a probable benefit in submaximal prolonged activity [70].

During activity, amino acid oxidation increases, and there is a general reduction of protein synthesis with a consequent increase in protein catabolism. Some amino acids, like alanin, are directly involved in gluconeogenesis. Others like leucine represent surrogate metabolic fuel for skeletal muscle, especially when lacking carbohydrates as a condition of restricted intake or in an insulin deficient state [71]. Even if protein catabolism minimally contributes to PA in terms of energy, correct intake of protein appears fundamental to enhancing recovery periods thus indirectly improving physical performance. People living with T1DM engaging in PA need 1.1–1.5 g of protein per kilogram of weight [72]. Strength training and long-duration aerobic training athletes must be particularly aware of the need to consume a sufficient amount of protein. After EXE consumption of 0.3 g of proteins per kilogram of weight is suggested. The remainder of the daily protein requirement could be distributed in 20–25 g portions every 3–4 h with the last at bedtime [73]. Generally, sources of proteins containing all essential amino acids (like milk, meat, and eggs) are preferable due to their better digestibility and capacity to significantly increase protein synthesis [74].

#### 3.3.3. Protein and Reduction of Hyoglycaemic Risk in T1DM Engaging in PA

Fear of hypoglycemia is one of the main barriers to PA in people living with T1DM. Reducing the insulin dose and administration of a certain amount of extra carbohydrates during or immediately after PA are two strategies to minimize hypoglycemic risk. These actions do not, however, always succeed in preventing hypoglycemia, particularly late-onset. Consuming protein could represent an efficacious choice to minimize late-onset hypoglycemic risk while at the same time contributing to the enhancement of muscle recovery and endogenous protein recovery [75]. Choosing the correct amount of protein to be consumed by people living with T1DM when engaging in PA represents one of the main areas of clinical research in this field.

### 3.4. Nutritional Management of People Living with T1DM Engaging in PA: Focus on Nutritional Supplements and Micronutrients

Nutritional management of elite athletes living with T1DM requires a complex approach combining, in a finalized manner, specific pathophysiological elements of physical EXE with good short and long-term glycemic control [76]. To achieve that, it is important to establish an athlete’s nutritional needs in every phase of the athletic season, to indicate the right balance between insulin therapy and carbohydrate intake and use CGM to eventually correct acute alterations in blood glucose levels that can be dangerous for health. Dietary requirements can greatly vary due to an individual athlete’s characteristics such as genetics, age, gender, race, lifestyle, and sport-dependent characteristics including duration, intensity, frequency, and nature (aerobic, anaerobic, or mixed). For this reason, athletes living with T1DM should acquire specific education from qualified professionals such as diabetologists, sports doctors, and dieticians, about the main risks which may jeopardize health or performance and actions to be implemented during each specific situation (intense training period, a high-level competition, international sport events), even if not strictly connected with sporting practice.

Although many international guidelines underline the importance of a “food first” approach to fill the peculiar energy, macro, and micro-nutrient needs of the athlete with T1DM, its pathophysiological situation, and its variable nature may require the development of individualized and “easier” nutritional strategies including the conscious use of Nutritional Supplement [77].

According to the Australian Institute of Sport (AIS), supplements can be classified into an ABCD group classification scheme based on their evidence-based efficacy and security grade, as shown in Table 2.

The statement asserts that Group A and Group B supplements might be used by identified athletes within a supervised “Supplement Program”, according to shared evidence-based protocols, and that individual athletes may consider using them within specific research or clinical monitoring situations [74,75]. Regarding diabetes challenges, some of these approaches could be very helpful in supporting training sessions and competitions for people living with T1DM.

The reality, however, is different. According to the metanalysis of Hannon et al. [76], despite the widespread use of nutraceuticals in diabetes, many of these substances do not aid in long-term blood sugar control or have scientifically validated effectiveness. The only substances that have shown sufficient evidence in improving glycemic control were dietary fiber, selenium, and zinc [76]. We can suppose that some of these can help manage glycemic blood concentrations during PA, but further studies are needed to prove it.

Although there is a scarcity of studies of elite athletes with T1DM, the substances that so far have shown the most relevant evidence in this specific population are caffeine, beetroot juice/nitrate, and zinc.

A Canadian RCT study carried out by Zaharieva et al. analyzing the “possible role of caffeine in reducing episodes of hypoglycemia in patients suffering from T1DM during moderate and vigorous-intensity aerobic sports practice” concluded that caffeine capsule intake in doses of 6 mg/kg before sports practice is effective at reducing hypoglycemia during aerobic EXE modestly but can increase the risk of hypoglycemia in the subsequent 24/48 h post-EXE [77]. Dietary nitrate, found in vegetables as NO (Nitric Oxide) 3-, is converted to NO2- and NO in the body. It has been linked to improved blood flow and oxygen demand during EXE through vasodilation. Studies investigating the effects of nitrate supplementation in individuals with T1DM have shown varying results due to inconsistent methodologies. However, we can suppose that higher NO blood concentration can help maximize muscle metabolism. Further studies are needed to justify routine supplementation. However, general dietary recommendations including a diet rich in fruits and vegetables such as spinach, red beets, and lettuce should be encouraged and may result in lower oxygen demand during suboptimal labor [78]. A recent randomized controlled trial meta-analysis conducted by Wang et al. discovered that individuals with higher zinc concentrations showed improvements in fasting glucose, fasting insulin, homeostasis model assessment for insulin resistance (HOMA-IR), and HbA1c. The conclusion drawn from the study suggested that zinc supplementation could be a useful adjunct therapy for preventing or managing diabetes [79].

## 4. Discussion

PA and EXE are both beneficial to T1DM management [80]. Unfortunately, the greatest risk carried by EXE for people with type 1 diabetes is hypoglycemia, which can be severe, during or several hours after exercising.

A correct nutritional program associated with adequate insulin therapy is the key elements of a good conditioning program for physical EXE in high-profile athletes suffering from T1DM. A dietary regimen completed with all the macronutrients and micronutrients necessary to meet the athlete’s individual needs, correctly measured and timed for every specific training period, is not always sufficient to guarantee effective support for high-profile sports practice and can be a challenge during a performance. In this context, CHOs are essential, because they are fuel for performance but are also required for hypoglycaemia prevention. In addition, adequate levels of hydration, proteins, and lipids are needed especially for prolonged and high-intensity activities.

Providing education on appropriate insulin dose adjustments for various types of EXE can be beneficial, but it may not always suffice. Certainly, recommending the use of continuous glucose monitoring (CGM) and insulin pump therapy instead of MDI can also be advantageous and should be considered for individuals managing diabetes during PA.

The use of individualized, programmed, and functional supplementation strategies could help the athlete living with T1DM to deal with the specific challenges of this pathological condition and might be associated with less effort and higher performance status. For these reasons, we highlight the necessity of further studies, especially randomized clinical trials (RCT), which examine specific sports dynamics and analyze the main critical issues in order to develop personalized nutritional strategies including supplements.

## Figures and Tables

**Table 1:** CHO and insulin requirement before, during, and after PA according to different type of PA and BG at baseline. (Based on the Consensus Statement by Riddel et al. [1], and the position Statement of the European Association for the Study of Diabetes (EASD) [49] and of the International Society for Paediatric and Adolescent Diabetes (ISPAD) [40,41].

	Before					During (Exercise Duration > 30′)				After (Exercise Duration > 30′)			
EXE	Glycaemia mg/dL (Arrow Trends)	CHO (g/kg)	Insulin Bolus	Basal Rate (CSII)	Food Choice	CHO (g/kg)	Insulin Bolus	Basal Rate (CSII)	Food Choice	CHO (g/kg)	Insulin Bolus	Basal Rate (CSII)	Food Choice
Aerobic	>270	0	−25%	−25%	High-GI food next to exercise (i.e., sugar, honey, corn syrup, non-diet juices, sports drinks, energy gels) Low- to moderate-GI foods 2 h before exercise	0	−25%	−25%	High-GI food (i.e., sugar, honey, corn syrup, non-diet juices, sports drinks, energy gels)	0.2	−25%	Regular	High-GI for recovery Low-GI maintain CHO availability and stable glucose levels Bedtime snack containing protein after intense or extended physical activity to prevent nocturnal hypoglycaemia
	180–270	↑ 0.0 → 0.0 ↓ 0.1–0.2	−50%	−50%		0.0 0.0 0.1–0.2	−50%	−50%		0.4	−50%	−20% in 6 h	
	126–180	↑ 0–0.2 → 0.3 ↓ 0.4.0.5	−50% −50–75% −75%	−50%		0–0.2 0.3 0.4–0.5	−50% −50–75% −75%	−50%		0.4	−50%/75%	−20% in 6 h	
	<126	Delay exercise, treat hypo	−75%	−80%		Stop exercise, treat hypo	−75%	−80%		0.6	−75%	−40%	
Mixed	>270	0	−25%	Regular	High-GI food next to exercise (i.e., sugar, honey, corn syrup, non-diet juices, sports drinks, energy gels) Low- to moderate-GI foods 2 h before exercise	0	−25%	Regular	High-GI food (i.e., sugar, honey, corn syrup, non-diet juices, sports drinks, energy gels)	0.2	Regular	Regular	High-GI for recovery (if necessary) Low-GI maintain CHO availability and stable glucose levels Bedtime snack containing protein after intense or extended physical activity to prevent nocturnal hypoglycaemia
	180–270	↑ 0.0 → 0.0 0.1–0.2	−50%	−25%		0.0 0.0 0.1–0.2	−50%	−25%		0.4	−25%	−20% in 6 h	
	126–180	↑ 0.0–0.1 → 0.2 ↓ 0.3–0.4	−50%	−25%		0.0–0.1 0.2 0.3–0.4	−50%	−25%		0.4	−25%	−20% in 6 h	
	<126	Delay exercise, treat hypo	−75%	−50%		Stop exercise, treat hypo	−75%	−50%		0.6	−50%	−40%	
Anaerobic	>270	0	regular	Regular	Low- to moderate-GI foods 2 h before exercise	0	regular	Regular		0.2	Regular	Regular	Low-GI maintain CHO availability and stable glucose levels Bedtime snack containing protein after intense or extended physical activity to prevent nocturnal hypoglycaemia
	180–270	↑ 0 → 0 ↓ 0	−25%	Regular		0 0 0	−25%	Regular		0.4	−25%	−20% in 6 h	
	90–180	↑ 0.0–0.1 → 0.2 ↓ 0.3–0.4	−25%	Regular		0.0–0.1 0.2 0.3–0.4	−25%	Regular		0.4	−25%	−20% in 6 h	
	<90	Delay exercise, treat hypo	−50%	−25%		Stop exercise, treat hypo	−50%	−25%		0.6	−50%	−40%	

Legend: EXE = Exercise; CHO = Carbohydrates; GI = glycaemic index. Glycemic trend arrows: ↑ = increasing; ↓ = decreasing; → = not increasing or decreasing.

**Table 2:** The ABCD Classification system ranks sports foods and supplement ingredients into four groups according to scientific evidence [75].

Groups	Supplements
Group A Strong scientific evidence for use in specific situations in sport using evidence-based protocols	Sport Foods: Sports Drink, Sports Gels, Sports Confectionary, Sports Bar, Electrolyte supplements, Protein Supplements Mixed Macronutrient Supplement (Bar, Powder, Liquid Meal); Medical Supplements: Iron, Calcium, Vitamin D, Multivitamin, Probiotics, Zinc; Performance Supplements: Caffeine, B-alanine, Bicarbonate, Beetroot juice/Nitrate, Creatine, Glycerol
Group B Emerging scientific support, deserving of further research.	Food Polyphenols: Fruit-derived polyphenols; Antioxidants: Vitamin C, *N*-Acetyl Cysteine; Tastants: Menthol, pickle juice, quinine; Others: Collagen Supplement, Curcumin, Ketone Supplements, Fish Oils (Omega 3), Carnitine.
Group C Scientific evidence not supportive of benefit amongst athletes	Magnesium, Alpha lipoic, acid HMB, BCAA/Leucine, Phosphate, Prebiotics, Vitamin E, Tyrosine.
Group D Banned or at high risk of substances that could lead to a positive doping test	https://www.wada-ama.org, accessed on 1 January 2024
